# *In Vitro* Cellular Adaptations of Indicators of Longevity in Response to Treatment with Serum Collected from Humans on Calorie Restricted Diets

**DOI:** 10.1371/journal.pone.0003211

**Published:** 2008-09-15

**Authors:** Joanne S. Allard, Leonie K. Heilbronn, Carolina Smith, Nicole D. Hunt, Donald K. Ingram, Eric Ravussin, Pennington CALERIE Team, Rafael de Cabo

**Affiliations:** 1 Laboratory of Experimental Gerontology, National Institute on Aging, National Institutes of Health, Baltimore, Maryland, United States of America; 2 Pennington Biomedical Research Center, Baton Rouge, Louisiana, United States of America; Universität Heidelberg, Germany

## Abstract

Calorie restriction (CR) produces several health benefits and increases lifespan in many species. Studies suggest that alternate-day fasting (ADF) and exercise can also provide these benefits. Whether CR results in lifespan extension in humans is not known and a direct investigation is not feasible. However, phenotypes observed in CR animals when compared to ad libitum fed (AL) animals, including increased stress resistance and changes in protein expression, can be simulated in cells cultured with media supplemented with blood serum from CR and AL animals. Two pilot studies were undertaken to examine the effects of ADF and CR on indicators of health and longevity in humans. In this study, we used sera collected from those studies to culture human hepatoma cells and assessed the effects on growth, stress resistance and gene expression. Cells cultured in serum collected at the end of the dieting period were compared to cells cultured in serum collected at baseline (before the dieting period). Cells cultured in serum from ADF participants, showed a 20% increase in Sirt1 protein which correlated with reduced triglyceride levels. ADF serum also induced a 9% decrease in proliferation and a 25% increase in heat resistance. Cells cultured in serum from CR participants induced an increase in Sirt1 protein levels by 17% and a 30% increase in PGC-1α mRNA levels. This first *in vitro* study utilizing human serum to examine effects on markers of health and longevity in cultured cells resulted in increased stress resistance and an up-regulation of genes proposed to be indicators of increased longevity. The use of this *in vitro* technique may be helpful for predicting the potential of CR, ADF and other dietary manipulations to affect markers of longevity in humans.

## Introduction

Prolonged calorie restriction (CR) produces beneficial health effects in many species including yeast, worms, flies, spiders, rodents, rabbits, dogs, monkeys and humans [1]–[4]. These benefits include improved insulin sensitivity, increased neuronal function and neurogenesis, enhanced stress resistance, decreased cancer incidence and in many species increased lifespan [5]–[8]. Some studies have suggested that alternate methods of energy manipulation, including alternate day fasting (ADF) and physical exercise, can produce many of the same health improvements observed with CR [9]–[14]. However, whether all these effects of CR extend to humans remains speculative. Reports on the dietary habits of the long-lived human population residing in Okinawa, Japan, suggest that CR may lead to an increased lifespan [15]. In addition, a two year study on CR in humans [16] reported many changes consistent with those found in lifespan studies of CR in rodents. Other evidence that CR may increase human lifespan can be found in ongoing studies showing that CR improves markers of disease risk and health in rhesus monkeys [17], [18]. Unfortunately, direct studies on the effects of CR on human lifespan are not feasible. However, the identification of biomarkers or indicators of increased longevity is a helpful tool in predicting the potential for CR to increase the lifespan and health-span of humans.

One proposed indicator of improved health and longevity is increased resistance to heat and oxidative stresses. With aging, there is a decline in tolerance to stressors such as heat and oxyradicals [19], [20]. In addition, there is a reduction in the expression of heat shock proteins [19]–[22]. Many of the beneficial effects of CR have been proposed to be mediated via heat shock proteins. Increased expression of heat shock proteins is related to increased heat tolerance, while a lack of these proteins induces intolerance to exposure to heat stress [23]. CR prevents these age-related declines in tolerance to heat and oxidative stress [20], [24], [25] and attenuates the reduction in expression of heat shock proteins [21].

Several important signal transduction pathways have been implicated in the regulation of the physiological processes of CR leading to increased lifespan. These pathways include the Sirt1 (the mammalian homolog of Sir2 (silent mating type information regulation 2)) pathway and the PGC-1α (peroxisome proliferator-activated receptor gamma, coactivator 1 alpha) pathway. In rodents, CR increases the expression of Sirt1 and PGC-1α in many tissues [26]. Sirt1 is an NAD(+)-dependent deacetylase which is considered to be important for the lifespan extending effects of CR. Sirt1 deacetylates a large number of transcriptional factors and cofactors involved in cell growth, differentiation, stress resistance, oxidative damage, and metabolism [26]–[31]. Sirt1 is also a regulator of PGC-1α [32], a transcription co-activator that plays a key role in regulating mitochondrial biogenesis, adipogenesis, muscle cell differentiation, and energy metabolism in multiple tissues. Reduced levels of PGC-1α are found in aged persons and in individuals with type-2 diabetes [33]. CR is known to attenuate age-dependent decreases in PGC-1α [34].

Many of the physiological effects observed in CR animals, including increased tolerance to heat and oxidative stress can be simulated at the cellular level by comparing cells cultured with media supplemented with blood serum obtained from animals fed a CR diet and those fed ad libitum (AL) [35]. This *in vitro* model of CR can be used to investigate mechanisms involved in the anti-aging effects of CR and may be beneficial in helping to predict the effect of various diets on the lifespan of humans.

In this study we utilized serum collected from participants of two pilot studies that were undertaken to examine the effects of ADF, CR and CR combined with aerobic exercise (CREX) on several markers of health and longevity in non-obese humans. *In vivo* effects from both studies have been presented in previous papers [2], [36]–[40]. We report here the results of the first study to assess the effect of human CR sera in cell culture experiments. In these experiments we compared human hepatoma cells (HepG2) cultured in media supplemented with human sera, obtained from participants 2 days before starting their diet regimen, to cells cultured with sera collected from participants at the end of their diet regimen. We then assessed the differences on cell growth, stress resistance, expression of Sirt1 and Hsp70 proteins and PGC-1 mRNA levels.

## Materials and Methods

### Human subjects and serum

Human serum samples, collected from a study of alternate day fasting in humans (the FEAST study) as well as the Comprehensive Assessment of the Long Term Effects of Reducing Intake of Energy (CALERIE) study, were provided by the Pennington Biomedical Research Center. Participants from each study were recruited as previously described [2], [38] and provided their written informed consent. The studies were approved by the Pennington Biomedical Research Center Institutional Review Board and the Data Safety Monitoring Board of CALERIE.

Participants of the FEAST study consisted of healthy, non-obese men and women (body mass index range: 20.0–30.0) between ages 23 and 53 years. The subjects had different levels of physical activity: 7 were sedentary, 3 were moderately active (exercised 1–2 times/wk), and 6 were highly active (exercised 4–5 times/wk). Competitive athletes and subjects with type-2 diabetes were excluded. The subjects attended the clinical research center on 2 consecutive days at baseline during which time baseline measurements and blood samples were taken. After baseline testing was completed, the subjects fasted from midnight to the subsequent midnight on alternating days for 21 days. On each fasting day, the subjects were allowed to consume energy-free beverages, tea, coffee, and sugar-free gum and were instructed to keep their water intake high. On feasting days, the subjects were instructed to eat whatever they wished and were informed that doubling their usual food intake would be required to maintain their usual body weight. Both baseline (2 days before beginning the diet regimen) and end-of-diet (day 21) serum samples used for this study were collected after a 12 h fasting period.

Participants of the CALERIE study included healthy, overweight (BMI = 25–30) males and females aged 25–50 and 25–45 years respectively. Participants were randomized into 1 of 3 groups: control weight maintenance diet (C), 25% calorie restriction from baseline energy requirements (CR), and 12.5% calorie restriction plus 12.5% increase in energy expenditure by structured exercise on a stationary bike or treadmill 5 days per week (CREX). All diets were based on the American Heart Association recommendation (≤30% of calories from fat). Serum samples collected at baseline and after 3 months of dieting were used for this study. All samples were collected after an overnight 12 h fasting period. Serum samples from 9 C, 11 CREX, 11 CR and 11 ADF participants were used in this study. Serum samples were thawed and heat inactivated at 56°C for 30 min. prior to use in cell culture experiments.

### Cell culture

The HepG2 human hepatoma cell line was used in these experiments because they are a commonly used, well characterized and standardized cell line which is known to express many of the genes of interest. In addition, our previous work utilizing this *in vitro* model, with serum collected from CR rats, was done on a similar rat hepatoma cell line (Fao) and we were interested in mimicking those results using human serum on a human hepatoma cell line. HepG2 cells were cultured in RPMI medium supplemented with 10% fetal bovine serum (FBS) and 1% antibiotics (Gibco, Gaithersburg, MD) until the time of treatment. Cells were cultured in a humidified incubator with 5% CO_2_, 95% air, at 37°C. For treatment, FBS was replaced with human serum from participants of the FEAST or CALERIE study. Cells cultured with serum collected from each individual at baseline (2 days before the start of the diet regimen) were compared to cells cultured with serum collected from that same individual at the end of the dieting period.

### Cell proliferation and survival measurements after exposure to heat shock and hydrogen peroxide

HepG2 cells were obtained from ATCC (Rockville, MD). HepG2 cells were seeded at a density of 10,000 cells per well into 96 well plates with media supplemented with human sera. Plates were separated into those treated with baseline sera and those treated with sera collected at the end of the dieting period from the same individual. The effect of serum from individual participants was evaluated separately. After 24 h, freshly prepared hydrogen peroxide (H_2_O_2_) was added to plated cells. Cells were challenged with either H_2_O_2_ (0–0.45 mM) for 24 h, 1 h incubation at 45°C, or left unchallenged for control. 24 hours after treatment, media was replaced with serum-free media and cell viability was determined by the addition of a tetrazolium salt solution, WST-8 (Dojindo, Indianapolis, IN) to each well according to manufacturer's protocol. WST-8 is bioreduced by cellular dehydrogenases to an orange formazan product that is soluble in tissue culture medium. The amount of formazan produced is directly proportional to the number of living cells. After 3 h of incubation, absorbance levels were measured at 450 nm using a microplate spectrometer. Absorbance levels were then translated to the viable cell number using a previously prepared calibration curve. Cell viability was calculated as the ratio of viable cells, after a given treatment, compared to untreated cells.

### Western blot analysis of Sirt1 and HSP70

HepG2 cells were cultured in RPMI media supplemented with 10% serum from human subjects for 48 h. Plates were separated into those treated with baseline sera and those treated with sera collected at the end of the dieting period from the same individuals. Whole cells were lysed in ice-cold RIPA buffer (1× PBS, 1% Igepal, 0.5% sodium deoxycholate, 0.1% SDS) with freshly added protease inhibitors (Protease inhibitor cocktail P8340, Sigma-Aldrich, St. Louis, MO; Phenylmethylsulfonylfluoride (PMSF), Fluka-Biochemica, Switzerland), incubated on ice for 30 min and centrifuged at 14,000 *g* for 10 min at 4°C. After centrifugation, the supernatant of the HepG2 cell lysate was collected and total protein concentrations were measured by the Bradford method. Whole cell lysate was added to 5× electrophoresis sample buffer and separated by SDS/PAGE under reducing conditions on a 12% separation gel. Proteins were transferred to PVDF membranes (16 h, 4°C). Unspecific binding was blocked by incubation in 5% milk blocking buffer (1× PBS, 5% nonfat milk and 0.1% Tween 20). Membrane bound proteins were then immunoblotted with antibodies to actin, HSP70 (Santa Cruz Biotech, Santa Cruz, CA), or Sirt1 (Millipore, Billerica, MA). Signals were developed using ECL reagent (Amersham Pharmacia Biotech, Buckinghamshire, England) and densities of the bands were evaluated using a Syngene Gene Genius Bio-Imaging System (Imgen, Alexandria, VA). Protein loading was evaluated either using β-actin antibody (Santa Cruz) or Ponseau S staining (Sigma-Aldrich).

### PGC-1α real time RT-PCR

HepG2 cells were cultured in 10% human sera, collected at either baseline of at the end of the dieting period, for 48 h. The effect of serum from individual participants was evaluated separately. Total RNA was extracted using an RNA isolation kit (Zymo Research, Orange, CA) according to the instructions of the manufacturer. The concentration and the purity of RNA were determined by spectrophotometry. 500 ng of RNA were reverse-transcripted using The SuperScript III First Strand Synthesis System (Invitrogen, Carlsbad, CA) according to the manufacturer's instructions. Real-time PCR reactions were performed in a 50-µl mixture containing 2 µl of cDNA, 10 µl of SYBR Green (Super Array, Frederick, MD), and 5 µM of human PGC-1α primers (forward: tccttgcagcacaagaaaac and reverse: gtctgcttcgtcgtcaaaaa) and human GAPDH Primers (forward: caaggagtaagacccctgga and reverse: gggtctacatggcaactgtg) on a 7300 Real Time PCR system (Applied Biosystems, Foster City, CA). The absence of nonspecific products was confirmed by the analysis of the melting point curves and by electrophoresis in 1.5% agarose gels. GAPDH served as an internal standard of mRNA expression. mRNA quantification was achieved by constructing standard curves using cDNA of known concentrations. The standard curves consisted of serial dilutions of the PCR amplicon from HepG2 cells, corresponding to the gene of interest and quantified using a spectrophotometer (expressed as ng/µl). Four standard samples were included in each run. All concentrations of target gene cDNA were calculated relative to their respective standard curves. All samples, including controls and standards, were done in triplicate to control for discrepancies in pipetting.

### Statistical analysis

Significant differences between cells treated with serum samples collected at baseline and serum samples collected, from the same individual, after 3 months on the CALERIE regimens or after 3 weeks on the FEAST regimen were assessed by the paired *t*-test. *P*<0.05 was considered statistically significant. A one-way ANOVA was used to determine any differences in the magnitude of change between cells treated with serum from different diet group participants. When a significant effect was found, differences between means were determined by Fisher's Probability of Least Significant Difference post hoc test.

## Results

### HepG2 cells cultured with serum from participants after 21 days of ADF (FEAST) showed a decrease in cellular proliferation and increased heat shock viability compared to cells cultured with baseline serum

**Figure 1:**
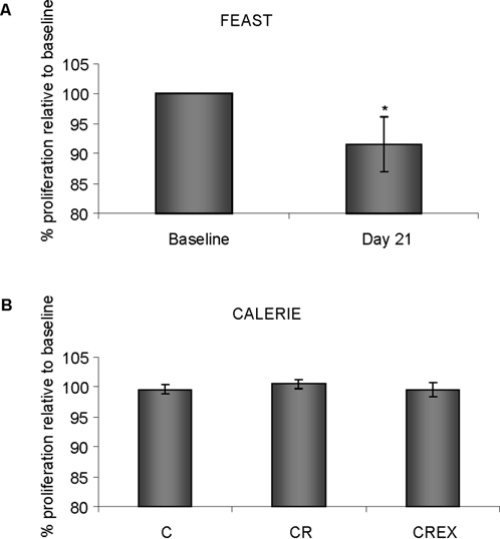
Proliferation of HepG2 cells cultured with serum from FEAST and CALERIE participants. HepG2 cells were cultured in 96 well culture plates in culture media supplemented with 10% human serum. After 48 hours in culture, differences in the proliferation of HepG2 cells in serum collected at day 21 from FEAST participants or after 3 months from CALERIE participants were compared to the proliferation of cells cultured in serum from the same participants collected on baseline day. Graph shows % proliferation relative to baseline of cells cultured in serum collected from participants of the (A) FEAST study (n = 11) and (B) CALERIE (n = 9 for C group, n = 11 for CR group, n = 11 for CREX group) study. * = statistically significant p = 0.032).

Proliferation of HepG2 cells cultured in serum collected at day 21 from participants of the FEAST study showed a 9.3% decrease (p = 0.032) compared to cells cultured in serum collected at baseline (Fig. 1). There were no differences in cell proliferation between cells incubated in serum collected at baseline or after 3 months of CR or CREX from CALERIE participants (Fig. 1).

**Figure 2:**
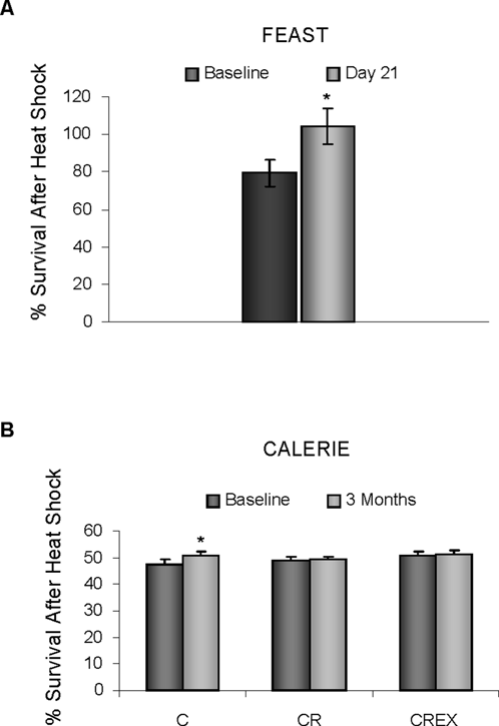
Resistance to heat shock of HepG2 cells cultured with serum collected at baseline and serum collected at the end of the diet regimen from FEAST and CALERIE participants. HepG2 cells were cultured for 24 hrs in 96-well culture plates in culture media supplemented with 10% human serum. Plates were then either subjected to a 1 hr incubation period at 45°C or left under normal culture control conditions. 24 hrs after heat shock treatment, surviving cells were determined using a CCK-8 soln. Graph shows an increased resistance to heat shock in cells cultured with FEAST serum, * = p<0.0005 (A) and no changes in cells cultured in serum form all groups of the CALERIE study (B).

Cells cultured with day 21 serum from FEAST participants showed a significant, 25% increase in survival 24 h after a 1 h heat shock treatment (Fig. 2) as compared to cells cultured in baseline FEAST serum (p<0.0005). Cells cultured in baseline FEAST serum had a 79.4% survival rate compared to the non heat-shocked baseline cultures. Cells cultures in day 21 FEAST serum showed complete tolerance and proliferation in response to the 1 hr. heat shock. Cell numbers reached 104.4% that of the non-heat-shocked day 21 cultures.

One hour of heat shock treatment caused cell survival to drop by an average of 50% in all cells cultured with 3 month serum from the CALERIE study. No differences were found between baseline serum treated and 3 month serum treated cultures from CR and CREX groups. However, there was a small (6.8%) but significant increase in survival of cells treated with 3 month serum from the control group, compared to baseline serum treated cells (p = 0.031). There was no significant difference between CALERIE groups.

Exposure to increasing concentrations of H_2_O_2_ resulted in a decreasing survival rate of cells. However, there were no differences in viability between cells cultured with baseline serum and those cultured in serum collected after 3 months of CR or CREX or 3 weeks of ADF (data not shown).

### Sirt1 expression was increased in cells incubated with serum from participants in the FEAST study and the CR group of the CALERIE study

**Figure 3:**
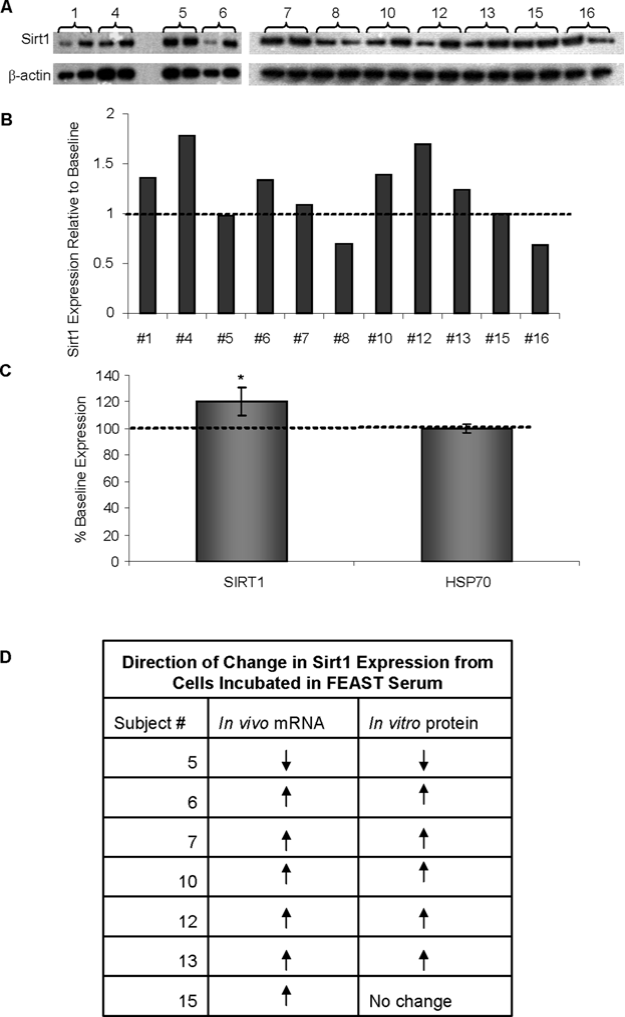
Increased Sirt1 protein levels in cells incubated in FEAST sera. Sirt1 expression was evaluated using western blotting techniques. (A) The first and second band of each pair of bands under the subject # represents relative levels of Sirt1 protein induced by baseline and day 21 sera samples respectively. (B) shows relative values of levels induced in cells cultured in day 21 sera with respect to cells cultured in baseline sera. (C) Cumulative average change in Sirt1 and HSP70 levels induced by day 21 sera with respect to baseline sera. *p<.0005 (D) shows the directional change in Sirt1 mRNA levels in muscle biopsies from baseline to 3 weeks matched exactly in 6 out of 7 participants whose serum was used for Sirt1 protein *in vitro* analysis.

**Figure 4:**
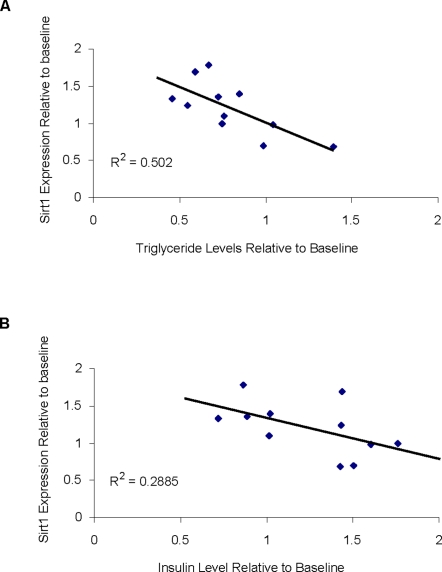
Correlations of changes in Sirt1 protein expression with changes in serum insulin and triglyceride levels with respect to baseline levels. Changes in Sirt1 protein expression from cells cultured with baseline and end of diet serum from individuals of the FEAST study were correlated to changes in each individual's serum levels of triglycerides (A) and insulin (B) relative to baseline levels.

A 20% increase in Sirt1 protein levels (p<0.05) was observed in cells treated with serum obtained from FEAST participants following 3 weeks of ADF compared to cells treated with baseline serum (Fig. 3). A previous paper showed changes in Sirt1 mRNA levels in muscle biopsies taken from the same FEAST participants [37]. Figure 3D shows that the directional change in Sirt1 expression found in this *in vitro* study was generally consistent with the changes in muscle mRNA levels from a subset of participants. Correlations between the changes in Sirt1 protein levels induced in HepG2 cells cultured in day 21 versus baseline FEAST serum and the changes in serum levels of triglyceride (r = −0.708, p = 0.015) and insulin (r = −0.537, p = 0.088) are shown in Figure 4.

**Figure 5:**
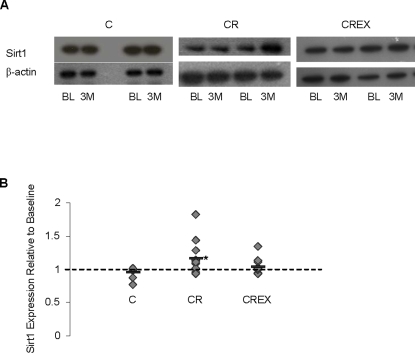
Increased Sirt1 protein levels in cells incubated in CR sera. Sirt1 expression was evaluated using western blotting techniques. (A) Shows representative blots of Sirt1 and β-actin protein. The first and second bands of each pair of bands represent relative levels of Sirt1 protein induced in HepG2 cells by baseline (BL) and 3 month (3 M) sera samples, respectively. (B) Shows changes in sirt1 levels relative to baseline. For C, CR and CREX n = 9, 11 and 11 respectively. * p = 0.018.

Sirt1 protein levels from cells cultured in serum collected after 3 months of diet were an average of 96.2%, 117.3% and 104.4% of the protein levels of cells treated with baseline serum from C, CR, and CREX participants respectively (Fig. 5). The changes in Sirt1 protein levels was significant in the CR group (Fig. 5, p = .013), but not the C or CREX groups. There were no differences in HSP-70 protein levels in HepG-2 cells cultured with baseline serum versus intervention serum in FEAST or CALERIE participants.

### PGC-1α mRNA levels were increased in cells incubated with serum from participants on CR

**Figure 6:**
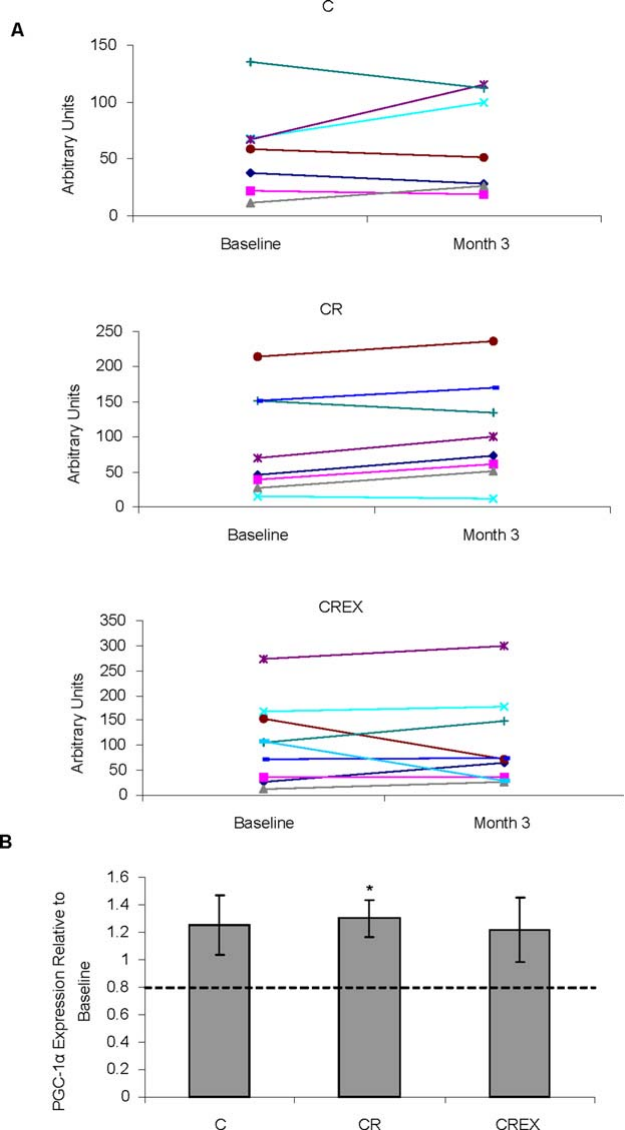
Changes in PGC-1α mRNA expression in cells cultured with sera from CALERIE Participants. HepG2 cells were cultured in media supplemented with human sera for 48 h (A) shows relative changes in PGC-1α mRNA in cells cultured with serum collected after 3 months on diets compared to cells cultured with serum collected at baseline. (B) Shows the average relative change in PGC-1α mRNA expression for each diet group. For C, CR and CREX n = 7, 8 and 9, respectively. * p = 0.032.

PGC-1α mRNA was isolated from HepG2 cells that were cultured for 48 h in baseline serum and 3 month serum from participants of the CALERIE study. Results for cells cultured with individual serum samples are plotted in Figure 6. Although all groups showed an average increase in expression and there was no difference in mean change across the groups, the increase in PGC-1α mRNA expression in cells cultured in serum from the CR group was significantly different from baseline (p = 0.03). Cells incubated in C and CREX serum showed no significant changes in PGC-1α mRNA expression from baseline.

## Discussion

In 2003, de Cabo and colleagues described an *in vitro* technique demonstrating that many of the features of CR including reduced cellular proliferation, enhanced stress responsiveness and changes in gene expression could be reproduced in cells cultured in media supplemented with serum from animals on CR diets [35]. These findings suggested that several effects of CR are mediated through circulating factors in the sera of the animals subjected to the dietary regimen. In fact, studies have shown that changes in hormones and nutrients that are constituents of serum including insulin, glucose, IGF-1 and fatty acids, modulate gene expression pathways involved in stress responsiveness and longevity [41]–[44]. Since it is unlikely that long-term studies determining the effect of CR or other dietary manipulations on human lifespan will be conducted, this *in vitro* technique is extremely useful for the investigation of changes in biomarkers of aging and longevity, which may be predictive of effects on lifespan. Therefore, using this *in vitro* technique, we examined the effect of human serum samples collected from FEAST and CALERIE participants on cellular proliferation, stress resistance and protein expression.

We found that changes in stress resistance as well as mRNA and protein levels of genes considered to be important for the beneficial effects of CR can be induced in cells incubated in media supplemented by serum collected from human participants who had been adhering to different CR regimens when compared to cells incubated with serum from the same individuals before the dietary manipulation. Serum collected at the end of the diet period from participants on the ADF regimen induced significant decreases in proliferation, increases in heat shock resistance and increased Sirt1 levels compared to baseline serum, while serum samples from participants on the CR regimen also induced significant increases in Sirt1 levels in addition to significant increases in PGC-1α mRNA expression compared to baseline serum. These findings are supportive of improved health and longevity.

Long term CR is known to decrease cellular proliferation in many tissues, which is associated with lower cancer risk [45], [46]. In addition, CR has been shown to have protective effects against oxidative and heat stress [20]. In this study, we observed that human hepatoma cells, cultured with blood serum collected after 21 days of ADF, have a decreased rate of cellular proliferation and an increased resistance to heat-induced stress, but not to oxidative stress induced by H_2_O_2_, when compared to cells cultured with blood serum collected at baseline. Similar findings were reported previously which demonstrated that the treatment of cells cultured in sera collected from rats and monkeys fed CR diets cause reduced cell proliferation, enhanced tolerance to heat and oxidative stress and an increased expression of stress response genes when compared to cells cultured in AL sera [35]. Cells treated with serum from CALERIE participants on CR and CREX regimens for 3 months, however, showed no significant changes in cellular proliferation or heat shock resistance compared to cells cultured in the baseline serum. Although FEAST participants were instructed to eat twice their usual intake on feast days in order to maintain their bodyweight, subjects lost an average of 2.0% of their initial bodyweight by the end of the 3 week period. In comparison, CR and CREX subjects lost respective averages of 7.4% and 5.8% of their initial bodyweight after 3 months on their diet regimens. Therefore, overall weight loss was not the necessary impetus that led to these effects on proliferation and heat stress resistance. Instead, it may be that the short, regular intervals of complete caloric deprivation, as practiced by FEAST participants, provided a more potent stimulus for triggering the necessary changes in serum constituents than either the CR or CREX regimens practiced by the CALERIE participants. Indeed, other studies in rodents have shown that ADF results in increased stress protection, sometimes surpassing the protective effect seen with CR [9], [47]. In addition, FEAST subjects showed several phenotypes, typical of CR, which may play a role in the stress response, including decreased insulin and triglyceride levels [38]. A surprising outcome was the small but significant increase in survival after heat shock treatment seen in cells cultured with 3 month serum from CALERIE control subjects compared to baseline serum treated cells. Further analyses will be needed to provide a possible explanation for this outcome since control subjects showed no changes in the neuroendocrine measurements taken that may provide a plausible explanation. Changes in other factors that were not measured in CALERIE participants may have contributed to this unexpected effect. Although control group participants were not calorie restricted, their diet during the study provided a healthy 30% of calories from fat with the recommended daily allowance of nutrients. This regimen may have been very different from their usual diet and therefore may have induced changes leading to our finding. Also due to their healthier diet, they showed a tendency toward less DNA damage as reported previously [2]. The increase in heat shock resistance seen in cells cultured with FEAST serum was not accompanied by increased HSP70 protein levels as anticipated. However, other heat shock proteins not examined in this study (due to limited availability of serum samples) such as HSP90 or HSP72 may have been responsible for this protective effect. In addition, changes in resistance to oxidative stress were not seen in any of the human serum treated cells. Increased length of time on diet may be needed to induce the changes necessary to enhance resistance to oxidative stress in cells treated with the serum.

PGC-1α mRNA levels were examined in cells incubated with serum from CALERIE participants after 3 months on their diet regimens compared to cells cultured in baseline serum. Only cells cultured in serum from subjects on the CR regimen showed significant increases in PGC-1α mRNA expression. This result falls in line with those of Lopez-Lloyd and colleagues who reported that other members of the PGC-1α pathway including peroxisome proliferation-activated receptor (PPAR)α and nuclear respiratory factors (NRFs), showed increased expression in primary rat hepatoma cells and Fao cells cultured in serum collected from CR-fed rats compared to cells cultured in serum from AL-fed rats[48]. The increase in PGC-1α levels and other proteins of the pathway is associated with increased mitochondrial biogenesis and metabolic efficiency[48]. An increase in PGC-1α levels may have been expected to occur in cells treated with CREX serum since exercise is known to increase PGC-1α levels [49]. However, although exercise has been shown to be a potent stimulant for increased PGC-1α levels in muscle, it is not certain whether exercise also increases expression of this gene in other tissues including liver. If the changes in blood serum required to induce increases in PGC-1α in liver are capable of being induced by CR but not exercise, then the 12.5% CR in the CREX group may not have been adequate stimulus to produce the endocrine changes required to invoke PGC-1α.

Our finding that Sirt1 protein levels were increased in cells incubated with serum from participants on ADF and CR regimens is particularly interesting. Increases in Sirt1 protein is thought to be a major contributing factor for many of the health benefits associated with CR. Increased Sir2 levels have been associated with increased lifespan in yeast, flies, and worms [3], 4, 50 and Sirt1is a leading contender for proteins responsible for the longevity effect of CR in rodents [26], [51]. Sirt1 is an NAD-dependent deacetylase that activates and deactivates several proteins that regulate stress reactions, growth, and metabolism[52], [53]. In rodent models with extended lifespan through CR, an increase in Sirt1 levels is seen in many tissues including muscle and liver. Transgenic mice over-expressing Sirt1 mimic the physiologic responses of CR [54] and the Sirt1 activating molecule, resveratrol, has been shown to extend the lifespan of mice fed a high fat diet [55]. The increase in Sirt1 expression in cells cultured with serum from FEAST and CR CALERIE participants strongly corresponds with the increase in Sirt1 mRNA expression found in muscle biopsies from a subset of FEAST [2] and CR CALERIE participants [36].

The increased Sirt1 expression in our cells may suggest that ADF and CR induce changes in blood serum constituents that lead to increased resistance to stress. Previous studies have suggested that increased Sirt1 levels may be related to decreased fasting glucose and insulin levels and decreased body temperature [26]. In support of our findings with Sirt1, CR participants showed a decrease in core body temperature and fasting insulin levels [2]. FEAST participants showed decreases in fasting insulin and triglyceride levels but no changes in surface body temperature, although core temperature was not measured. In this study the observed changes in Sirt1 were significantly correlated to changes in serum triglyceride levels.

These *in vitro* results provide supporting evidence for the possibility of extending human lifespan and health-span through diet manipulations such as CR and ADF. In fact, our results indicate that ADF may have a more powerful effect than CR in inducing the changes in neuroendocrine factors that lead to increases in stress protection and Sirt1 protein levels. These are changes thought to be associated with improved health and increased longevity. In addition and importantly, this study lends support to the proposal that at least some of the effects of CR are partially due to changes in the hormonal and nutritive milieu of blood serum. In fact, a recent study investigating the age associated variations in immunity, showed that incubation of isolated peritoneal macrophages from young rats in sera collected from old rats results in a significant increase in inflammatory cytokines [56]. This and other similar *in vitro* studies further validate the use of this *in vitro* method to analyze changes in known and proposed indicators of health and longevity as well as the mechanisms behind the beneficial effect of CR. This *in vitro* method may also prove useful for predicting the potential of CR, ADF and other dietary treatments to affect human health and lifespan.
